# Alrecon: computed tomography reconstruction web application based on Solara

**DOI:** 10.12688/openreseurope.16863.1

**Published:** 2024-03-18

**Authors:** Gianluca Iori, Ibrahim Foudeh, Mustafa Alzu’bi, Malik Al Mohammad, Salman Matalgah

**Affiliations:** 1 SESAME - Synchrotron-light for Experimental Science and Applications in the Middle East, Allan, 19252, Jordan

**Keywords:** computed tomography, tomographic reconstruction, x-ray imaging, image processing, graphical user interface, Python, Solara

## Abstract

Synchrotron X-ray computed tomography is a non-destructive 3D imaging technique that offers the possibility to study the internal microstructure of samples with high spatial and temporal resolution. Given its unmatched image quality and acquisition speed, and the possibility to preserve the specimens, there is an increasing demand for this technique, from scientific users from innumerable disciplines. Computed tomography reconstruction is the computational process by which experimental radiographs are converted to a meaningful 3-dimensional image after the scan. The procedure involves pre-processing steps for image background and artifact correction on raw data, a reconstruction step approximating the inverse Radon-transform, and writing of the reconstructed volume image to disk. Several open-source Python packages exist to help scientists in the process of tomography reconstruction, by offering efficient implementations of reconstruction algorithms exploiting central or graphics processing unit (CPU and GPU, respectively), and by automating significant portions of the data processing pipeline. A further increase in productivity is attained by scheduling and parallelizing demanding reconstructions on high performance computing (HPC) clusters.

Nevertheless, visual inspection and interactive selection of optimal reconstruction parameters remain crucial steps that are often performed in close interaction with the end-user of the data. As a result, the reconstruction task involves more than one software. Graphical user interfaces are provided to the user for fast inspection and optimization of reconstructions, while HPC resources are often accessed through scripts and command line interface. We propose Alrecon, a pure Python web application for tomographic reconstruction built using Solara. Alrecon offers users an intuitive and reactive environment for exploring data and customizing reconstruction pipelines. By leveraging upon popular 3D image visualization tools, and by providing a user-friendly interface for reconstruction scheduling on HPC resources, Alrecon guarantees productivity and efficient use of resources for any type of beamline user.

## Plain language summary

Micro Computed Tomography (CT) is a widely known technology that employs X-rays to visualize the interior of a specimen with high resolution. 3D images are generated from CT scans via a computational process called CT reconstruction. Alrecon is a modern, open-source web application for CT reconstruction, designed keeping non-expert and new users of the CT technology in mind.

## Introduction

X-ray Computed Tomography (CT) is a powerful imaging tool mainly known for its impact in the medical field, where it allows healthcare professionals to visualize and analyze the internal anatomy in 3 dimensions, with high precision and clarity. CT scanners emit beams of X-rays that pass through the patient's body. X-ray detectors on the opposite side of the patient measure the intensity of the X-rays after they have passed through the body. Multiple X-ray projections are collected while rotating the X-ray source and detector around the patient. CT reconstruction refers to the computational process used to generate three-dimensional images of the internal structures of an object from sets of raw X-ray projections (also called sinograms).

Synchrotron X-ray Computed Tomography (SXCT) exploits the high brilliance and coherence of synchrotron radiation to provide tomographic microscopy of specimens with spatial and temporal resolution unmatched by medical or laboratory CT scanners. Users from a broad range of disciplines benefit from the capabilities of the several SXCT facilities (beamlines) installed at synchrotrons worldwide. During the normal operation of such laboratories, CT scans of a large variety of samples, with varying geometry and X-ray absorption properties, as well as a multiplicity of in-situ studies of materials can be performed. The process of tomographic reconstruction is performed routinely at SXCT beamlines to provide the users reconstructed volume images at the end of their beamtime and is a crucial step in data processing towards the extraction and publication of meaningful results from raw SXCT datasets.

Compared with laboratory CT, SXCT presents significant differences that are related to the different X-ray source nature, and to the different process of image formation. These differences are reflected in the process of data reconstruction, and in the range of artifacts that can affect SXCT sinograms. They also determine several opportunities that are unique to CT imaging with synchrotron light. The parallel geometry of collimated synchrotron beams implies that SXCT radiographs are generated as parallel projections of the object onto the image-forming, scintillator plane. Accordingly, the reconstruction process involves implementations of the Filtered-Back-Projection or other reconstruction algorithms based on a parallel geometry. On the contrary, image formation and tomographic reconstruction of laboratory or clinical CT are based on X-ray propagation of cone geometry. In SXCT radiographs, bright spots are generated by a variety of factors including dead or damaged detector pixels, dust, imperfections and impurities on the optical surfaces (e.g. mirrors, vacuum containment windows, lenses, scintillator) encountered by the beam along its propagation path, which can be up to hundreds of meters. In particular, the effect of dust and dirt on the scintillator surface is enhanced by the high resolution of the SXCT imaging system. In comparison and due to their large pixel size, the flat-panel detectors used in laboratory and clinical CT are less sensitive to the presence of impurities. In the sinogram space, this family of artifacts is described as stripes with a bright, saturated, or altered detector response. If not treated, such stripes give rise to ring artifacts in the reconstructed 3D image that can undermine image processing. Depending on the cause and features of stripe artifacts, these can be corrected using a variety of different algorithms before 3D reconstruction ^1^. Propagation-based phase-contrast X-ray tomography is an advanced imaging technique that exploits the unique coherence properties of X-ray illumination available at synchrotron facilities. Contrary to other approaches for phase-contrast imaging, propagation-based phase-contrast is generated without the need of dedicated equipment, by simply propagating the X-ray beam, after its interaction with the studied specimen, up to several meters before detection ^2^. Its straightforward implementation has made the technique an almost omnipresent feature of modern SXCT beamlines. Compared with standard, absorption-based CT, phase-contrast SXCT can achieve superior contrast to low or similarly X-ray absorbing materials, and a general reduction of the radiation dose deposited in the sample ^3^. The reconstruction of propagation-based phase-contrast tomograms requires an additional phase-retrieval step ^2^, which is applied to the sinogram prior to reconstruction. Another key advantage of CT with highly brilliant synchrotron radiation is the significant reduction of acquisition time, which enables time-resolved tomographic studies, with acquisition rates from a few Hz up to the kHz range ^4^. Time-resolved, in-situ SXCT has become an invaluable technique for observing in a non-destructive manner phenomena in 3D materials related to energy production and storage ^5, 6^, biomedical engineering ^7, 8^, geology and earth sciences ^9, 10^, and more. The drastic reduction of scan duration poses an additional challenge for CT reconstruction, since the time needed for dataset exploration and visualization can become the bottleneck in the experimental workflow. With data collection becoming faster, there is an increasing need for tools providing fast CT reconstruction and results visualization.

Due to the unique characteristics of synchrotron tomography, several open-source Python packages have been designed, in part or entirely, for the processing and reconstruction of SXCT datasets. These include TomoPy ^11^, TomoCuPy ^12^, Astra ^13^, Savu ^14^, and Nabu ^15^. These packages provide a range of tools and algorithms for sinogram manipulation, artifact correction, CT reconstruction, and post-processing operations. All these Python implementations of reconstruction software can be installed and scaled relatively easily (e.g. through app containers) for use on high performance computing (HPC) clusters allowing large-scale parallelization of processes across several central processing units (CPU), improving the 3D reconstruction performance dramatically. In addition, they allow enhancing the performance of particularly computationally intensive algorithms such as iterative reconstruction, by exploiting graphics processing unit (GPU)-accelerated versions of these. Despite extensive online documentation, the user of such packages is expected to be fluent in Python, as much as required by the customization and testing of complete reconstruction pipelines.

Toolkits such as Tofu ^16^ and STP ^17^ have been developed with the goal of providing user-friendly interfaces to CT reconstruction software. These are optimized for application on stand-alone workstations equipped with GPU. Dealing with large amounts of data and processes, such as those involved during a SXCT beamtime, using stand-alone equipment, presents several drawbacks:

- First, large chunks of experimental data must be transferred via local area network (LAN) to the reconstruction workstation. This introduces a workflow bottleneck and, in the worst case, can lead to data duplication. State-of-the-art SXCT laboratories, on the contrary, aim at eliminating experimental data copy and transfer. As an example, the data acquisition and computing infrastructure of the recently inaugurated beamline ID10-BEATS of SESAME, deploys high-throughput detectors able to generate massive experimental files on a centralized general parallel file system (GPFS) storage facility ^18^. The use of a hierarchical data format (HDF5), and 100 GBs connectivity between store and a hybrid HPC reconstruction cluster, eliminates redundancy and maximizes data transfer rates.
- Second, the necessity to inspect reconstructions immediately after the scan requires computing resources to be permanently available for data exploration and parameter optimization. Even utilizing fast GPU implementations of reconstruction routines, processing massive scans can take several minutes and hundreds of dedicated volatile memory, leading to inevitable multiplication of resources. In this context, a wiser allocation of resources seems to be a workstation dedicated to data exploration, while a queue of reconstruction jobs is handled by a job scheduling system such as Slurm ^19^ on the nodes of a HPC cluster.

### Statement of need

Alrecon (الريكُن), a user-friendly open-source Solara web application for SXCT data reconstruction with TomoPy is presented ^20^.

- In a single Python web application, Alrecon allows to inspect and optimize CT reconstructions interactively, and submit demanding reconstruction jobs to a HPC cluster, while keeping a flawless log of these operations.
- For 3D sinogram and reconstruction visualization, Alrecon leverages on napari and ImageJ, two powerful toolkits known by a vast community of users.
- The Solara framework makes Alrecon intuitive, while keeping the app robust and flexible.
- The modular, pure Python implementation of Alrecon allows expert users to scale and customize the app to their infrastructure.

Alrecon aims at bridging the gap between the large number of tools offered by reconstruction Python packages, and the routine exploration and processing of a large number and variety of SXCT datasets. The target user of Alrecon is the experienced SXCT beamline staff, as well as researchers or students lacking programming experience.

## Methods

### Implementation: Alrecon pipeline

**Figure 1.**
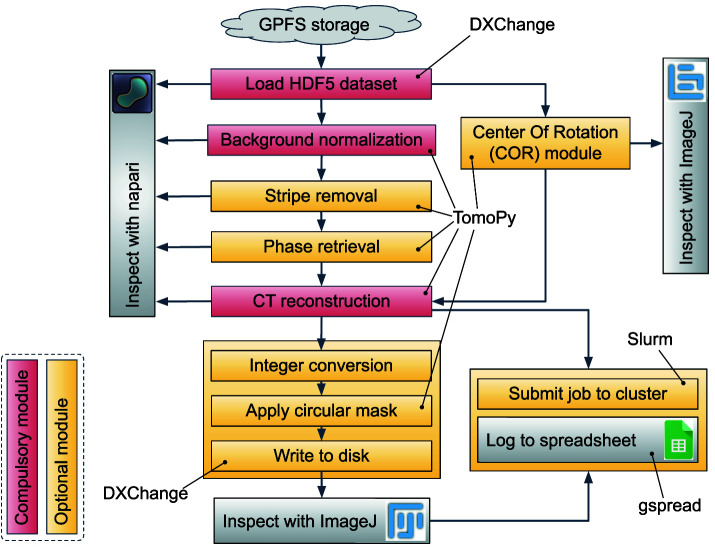
CT reconstruction pipeline implemented in Alrecon. Red: compulsory modules. Yellow: optional modules.

Figure 1 illustrates the pipeline for SXCT data reconstruction implemented by Alrecon. During the routine operation of the tomography beamline ID10- of SESAME, hierarchical HDF5 experimental files containing X-ray projections, reference flat and dark images, as well as a metadata archive of instrument and acquisition parameters, are loaded from a centralized GPFS short-term storage ^21^.

During a scan at BEATS, the raw experimental data (X-ray projections) is streamed by the detector directly to the central GPFS storage ^18^. Raw experimental data and metadata is written on a common HDF5 file that is generated by a data acquisition system on the GPFS storage, following the Scientific Data Exchange community standard ^22^. Experimental file duplication or modification after conclusion of the scan is not allowed.

**Pre-processing and reconstruction of sinograms.** The sinogram is loaded as a numpy 3-dimensional array and treated in different steps before reconstruction. Background normalization using the collected flat and dark frames can be generally performed automatically after loading the dataset. Optional modules allow to correct different types of sinogram stripes, and to retrieve the X-ray phase shift from absorption-contrast X-ray projections of the object. An Alrecon page dedicated to the optimization of the center of tomographic rotation (COR) parameter for the reconstruction is available. In most cases, the user is expected to inspect visually and confirm the choice of COR before proceeding to CT reconstruction. Once the COR parameter is found, and the sinogram pre-processed, the reconstruction procedure is launched. At any step of the pipeline, napari and ImageJ can be called to inspect the processed sinogram, or the results of COR optimization and CT reconstruction.

**Post-processing of reconstructed data.** Two basic operations can be applied before writing data to disk: (i) the dataset can be converted to 8-bit or 16-bit unsigned integer formats, and (ii) a cylinder masking filter can be applied to remove the corners of the 3D image. The best way to verify that the process was successful, and that all parameters are correct, is to inspect the final output with ImageJ.

**Launching reconstruction jobs on HPC cluster.** The pipeline described so far can be executed on a subset of the experimental file in just a few minutes (see next section for a detailed description). Once all parameters are optimized, a complete reconstruction job can be submitted to a HPC reconstruction cluster. At SESAME BEATS, the resources of one hybrid CPU-GPU HPC node equipped with 512 GB RAM are allocated permanently for this task during beamtime. In this way, a queue of large reconstructions can be handled on the main computing facility, taking advantage of the high-speed connection between GPFS storage and reconstruction cluster, while Alrecon remains available to the user for dataset and parameter exploration immediately after a new scan is completed.

***The Alrecon ecosystem*.** Alrecon’s functionalities are based on a set of open-source software and Python packages for web application reactiveness, data Input/Output, CPU and GPU tomographic reconstruction, process logging, and 3D image visualization. Table 1 summarizes the software ecosystem that supports Alrecon, with a description of the function provided by external software.

**Table 1.** Alrecon ecosystem.

Package/ Software name	Function	Reference
Solara	Solara is the backbone of the Alrecon application. It allows running Alrecon as a standalone, React-style web application, or inside Jupyter Notebook. Solara handles components state and reactiveness.	23
TomoPy	Python package for CT data processing and reconstruction. All sinogram pre-processing and reconstruction operations in the pipeline of Alrecon ( Figure 1) are implemented using TomoPy.	11
ASTRA	Toolbox for high-performance tomographic reconstruction methods on graphic processing units (GPUs). The ASTRA toolbox is accessed through its TomoPy integration.	13, 24
DXChange	Handles data Input/Output following the Scientific Data Exchange schema for HDF5 raw and metadata storage.	22
numpy	Core Python tool for N-dimensional arrays.	25
napari	Pure Python 3D image interactive viewer. Can be used to visualize the sinogram and reconstructed 3D images.	26
ImageJ	Multi-purpose image processing package. At SESAME BEATS, the Fiji distribution of ImageJ2 is used, which bundles several plugins for scientific image analysis.	27
gspread	Python API for Google Sheets.	
pandas	Used for reconstruction parameter logging.	28
Slurm	Workload manager used for reconstruction job scheduling on HPC.	19

### Operation: the Alrecon web application

**Figure 2.**
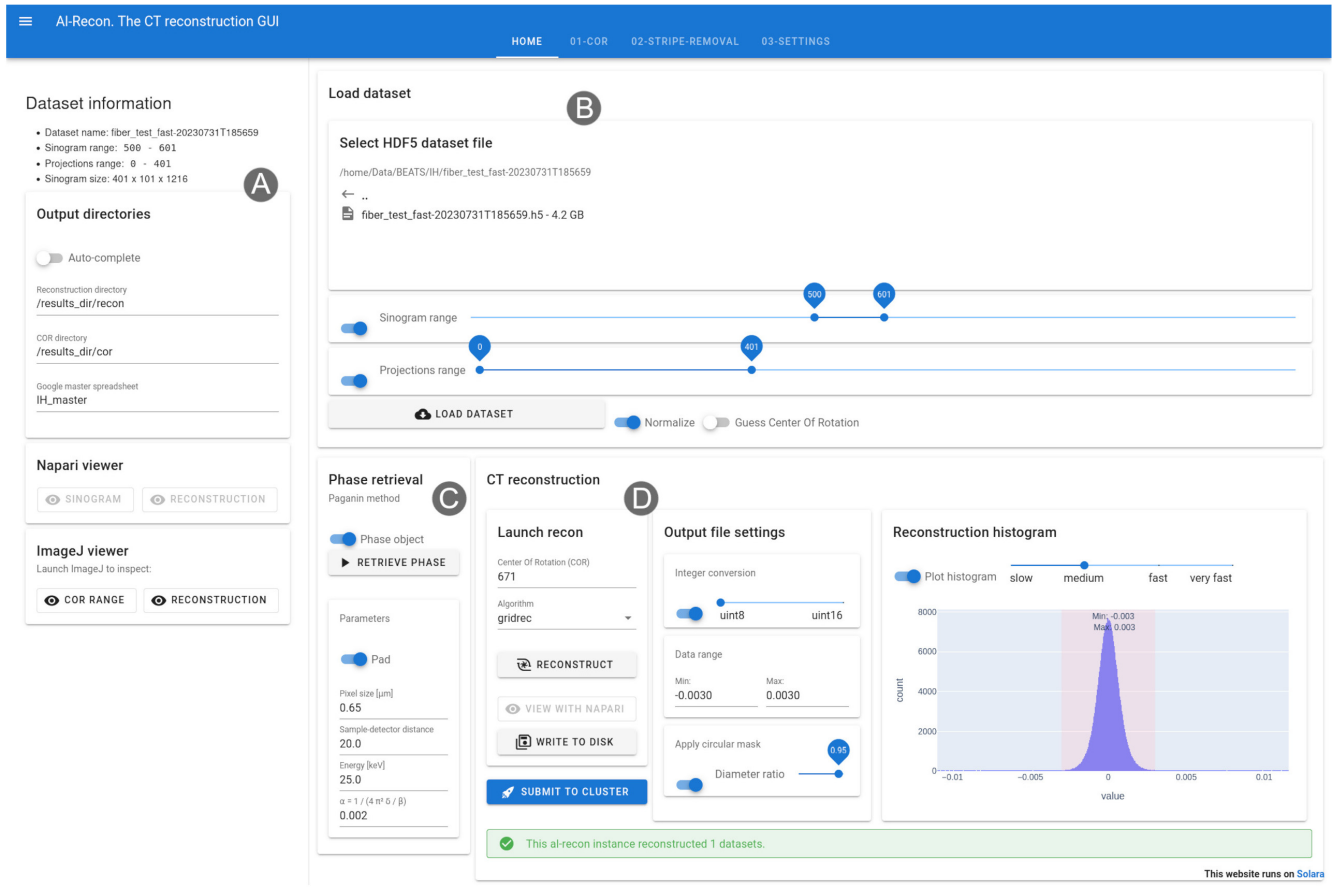
Home page of the Alrecon web application. ( **A**) Sidebar displaying dataset information and locations for output files and logs. ImageJ and napari can be launched from the sidebar. ( **B**) File load card. ( **C**) Phase retrieval card. ( **D**) CT reconstruction card.

The main page of the Alrecon web application is shown in Figure 2. On the left, a sidebar ( Figure 2-A) displays experimental dataset information, locations for writing of output files and the name of the beamtime master Google spreadsheet where reconstruction parameters will be logged. Two additional cards on the sidebar allow launching ImageJ and napari for visualizing results.

Experimental raw data stored in HDF5 files can be loaded as a whole or in chunks. The load dataset card ( Figure 2-B) allows the user to select sinogram (detector lines) and projections ranges before retrieving the data. Parameter optimization and reconstruction setup can be achieved in most cases on a subset of the sinogram. In this way, the time for file read and computation become practically negligible, and users experience an almost real-time visualization of results. This situation is desirable during the preliminary data exploration phase, in which several reconstruction attempts followed by visual checks of results are needed to find optimal parameters. Background normalization and a preliminary guess of the COR can be executed automatically after loading the data.

A phase retrieval card ( Figure 2-C) allows to process the sinogram and extract the phase shift induced by the specimen on the beam front, by applying the filter first described by Paganin *et al.* ^2^. Parameters describing the experiment setup required for the phase retrieval operation (i.e. object pixel size, sample-to-detector distance, and beam energy) are extracted automatically from the metadata archive associated with the loaded dataset. Normally, the user will tune the regularization parameter α:


$$\alpha \;=\;\;1/(4\pi^{2}\;\delta /\beta )$$

where *δ/β* is the ratio between the real and imaginary parts of the complex refractive index of the material.

The CT reconstruction card ( Figure 2-D) allows to launch and inspect the results of tomographic reconstruction processes. CPU and GPU implementations of several algorithms available in TomoPy and ASTRA can be selected. Conversion from floating-point to integer values, and a circular mask filter can be activated before writing the reconstructed dataset to disk. A plot of the intensity histogram of the reconstructed volume is used to select the optimal data range for representation as integers. Once the reconstruction settings are validated, a button allows the user to submit the reconstruction job on a centralized HPC cluster by performing the following:

1. A Slurm batch script file is written with reconstruction parameters taken from the active Alrecon session.
2. The batch script is submitted to Slurm on a dedicated HPC node.
3. The reconstruction parameters are logged to the master Google spreadsheet of the active beamtime.

**Figure 3.**
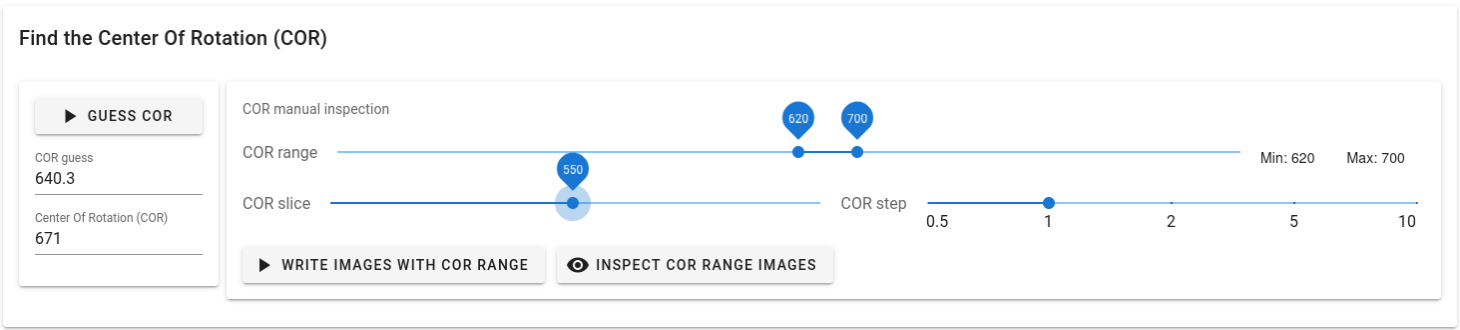
Center Of Rotation (COR) module. The page allows the user to find interactively the optimal COR parameter before CT reconstruction.

Alrecon’s module for optimization of the reconstruction COR is shown in Figure 3. Two methods are available for estimating the COR automatically ^11, 29^. The user can verify and optimize the choice of COR interactively, by inspecting a single slice of the 3D volume reconstructed with a range of COR values.

**Figure 4.**
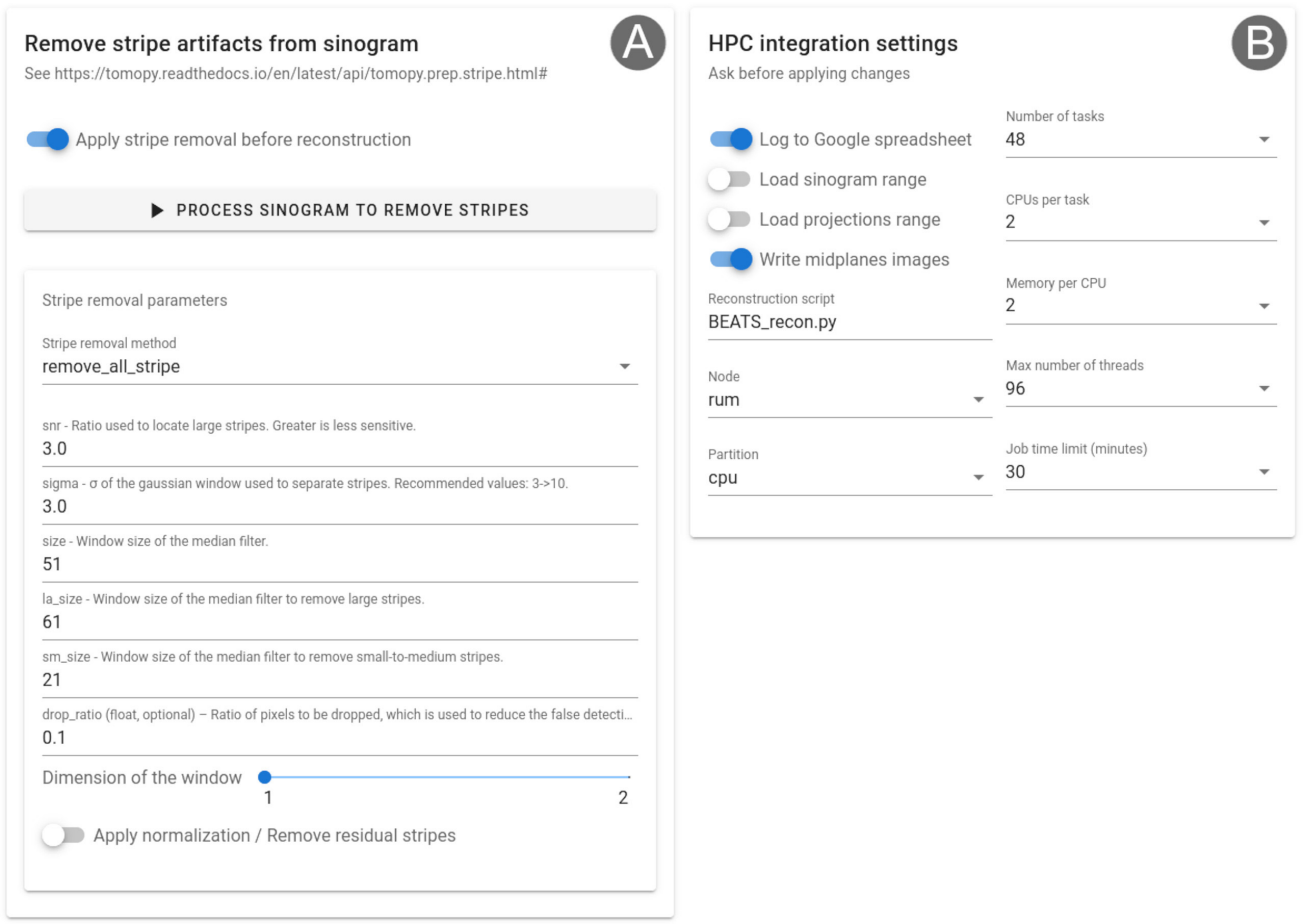
( **A**) Stripe removal module for the correction of stripe artifacts from the sinogram. ( **B**) Settings card for HPC integration, allowing to customize the behavior of Alrecon and Slurm job files written by the application.

The module for the correction of stripe artifacts from the sinogram is shown in Figure 4A. Four techniques implemented in TomoPy can be used to remove large stripes, fluctuating or unresponsive stripes caused by dead detector pixels, partial stripes, or a combination of methods ^1^. Figure 4B shows Alrecon’s HPC settings card, allowing expert users to modify parameters of the Slurm job scheduling and control.

## Use cases

This section demonstrates the functionalities described in the previous paragraphs, presenting results obtained at the ID10- beamline of SESAME using Alrecon. A Python environment for CT reconstruction with Alrecon was set up on a beamline workstation equipped with 36-core Intel Xeon W-2295 CPU @ 3.00GHz, 2× NVIDIA RTX A5000 GPUs, and 512 GB of RAM memory, following the installation instructions included in the home page of the Alrecon GitHub repository. The workstation is connected to the centralized GPFS storage facility through a 10 Gbps switch.

**Figure 5.**
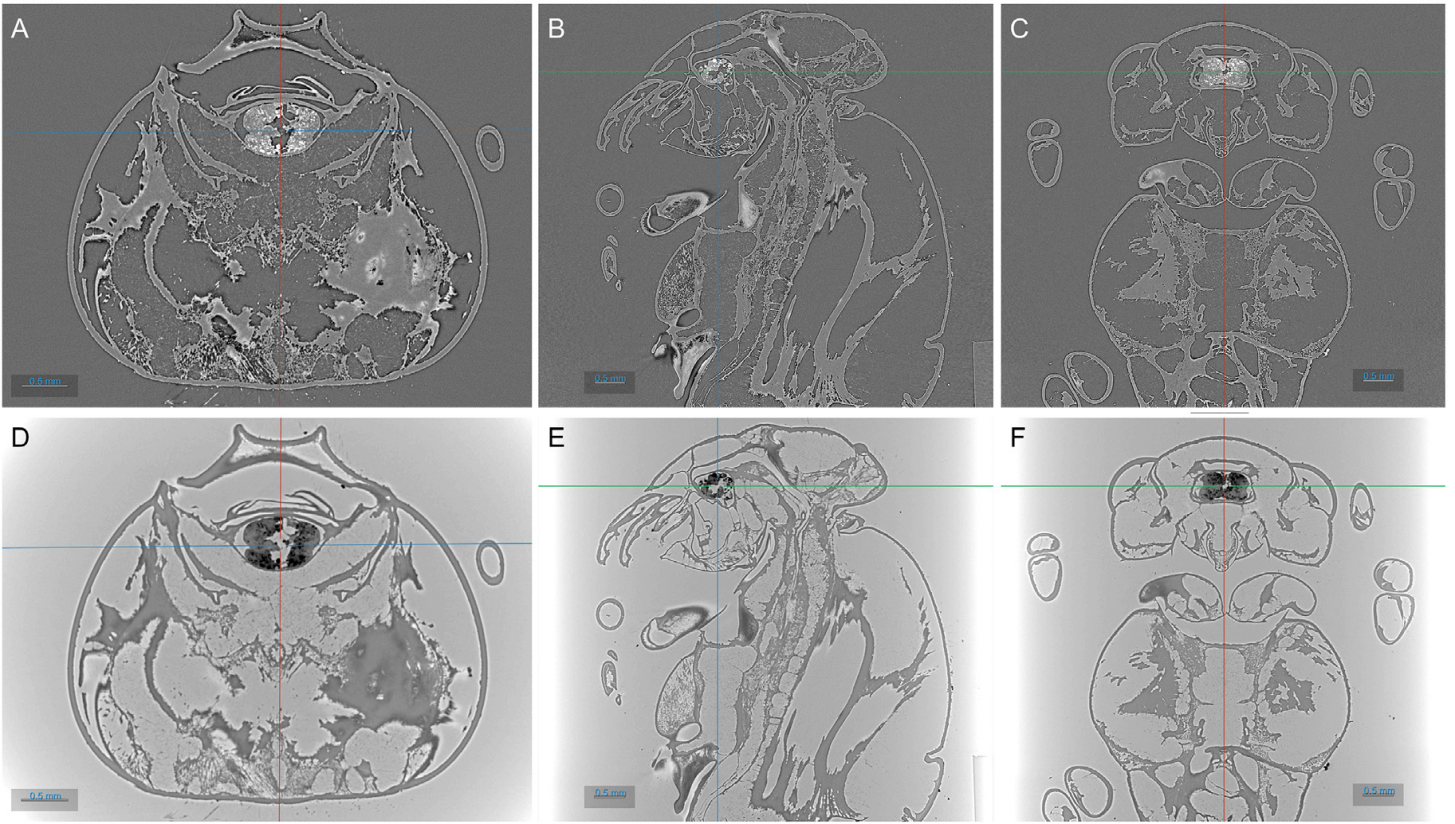
3D reconstruction of SXCT scan of a wasp performed at the ID10- beamline of SESAME. Top: absorption-contrast reconstruction. Bottom: phase-contrast reconstruction. From left to right: transverse, sagittal, and coronal planes through the insect’s body. Images were obtained with ORS Dragonfly.

Two cases of CT reconstruction performed with Alrecon are presented in Figure 5. Anatomical sections through the body of a dead wasp are shown. Figure 5A-C show the result of a standard, absorption-contrast image, while in Figure 5D-F the sample phase shift was reconstructed. SXCT scan and reconstruction settings are presented in Table 2. Reconstructions were performed on one of the GPU nodes of the beamline HPC cluster (2× Intel Xeon Gold 5220R @ 2.20GHz with 24 cores each, 576 GB RAM, Nvidia A100 40GB GPU), and can be reproduced downloading a repository of the raw data and the full metadata archive of the SXCT scan ^30^ (see the download link in the Data availability section). The online repository also contains a 3D video rendering of the reconstructed sample.

**Table 2.** SXCT scan and reconstruction settings for the wasp sample of Figure 5. The insect was dead when collected for scanning.

**Scan settings**	
X-ray beam modality	Filtered white beam
Energy (mean)	25 keV
N. Projections	2001
Exposure time	0.02 s
Scan modality	Continuous rotation - 360° rotation
Detector	PCO edge 5.5 sCMOS; 2560 × 2160 image format
Pixel size	3.1 μm
**Reconstruction settings**	
Center Of Rotation	1301
Stripe removal method	none
Algorithm	Filtered Back Projection (CUDA ASTRA implementation)
Filter	Ram-Lak
Output file format	8-bit unsigned integer
Floating point representation range	Absorption contrast: [-0.0015, 0.0015] Phase contrast: [-0.0006, 0.004]
Reconstructed image size	2560 × 2560 × 2160
**Phase retrieval settings**	
Algorithm	Paganin *et al.* ^2^ (TomoPy implementation)
Sample to detector distance	115 mm
α	0.0002

**Figure 6.**
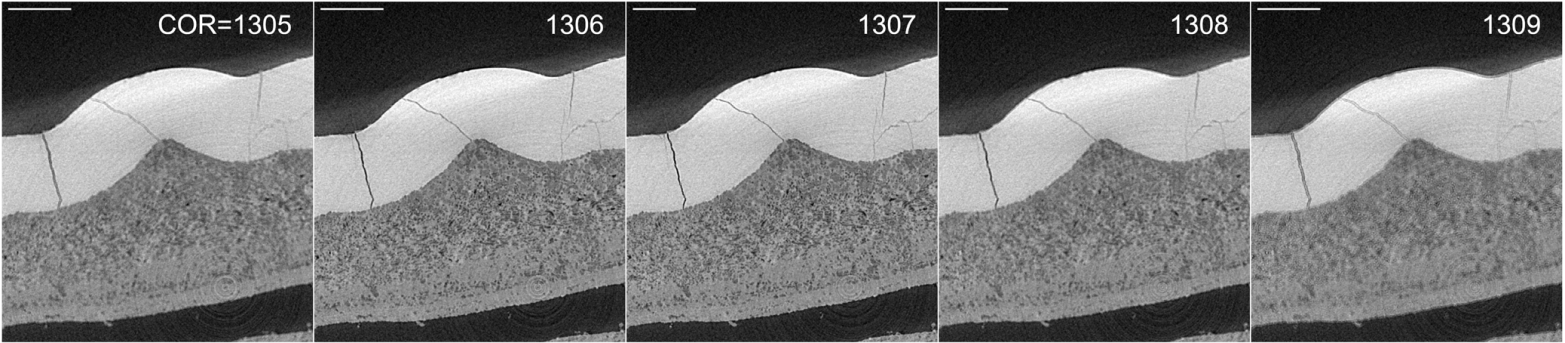
Slice (detail) of archaeological tooth scan reconstructed with different Center Of Rotation (COR). The image in the center (COR=1307) shows the better contrast and resolution of small features. After visual inspection of one slice, the whole dataset is reconstructed with COR=1307. Scale bar on the top-left of each image: 0.5 mm.

Figure 6 shows the result after optimizing the reconstruction COR for a SXCT scan of an archaeological incisive tooth sample. The detail of one axial slice through the tooth is shown, after reconstruction with different COR values. The reconstruction was performed with the Gridrec algorithm implementation of TomoPy ^11, 23^. Through the Alrecon application it is possible to reconstruct a slice with different COR values and identify the optimal COR by inspecting with ImageJ the result of the operation.

**Figure 7.**
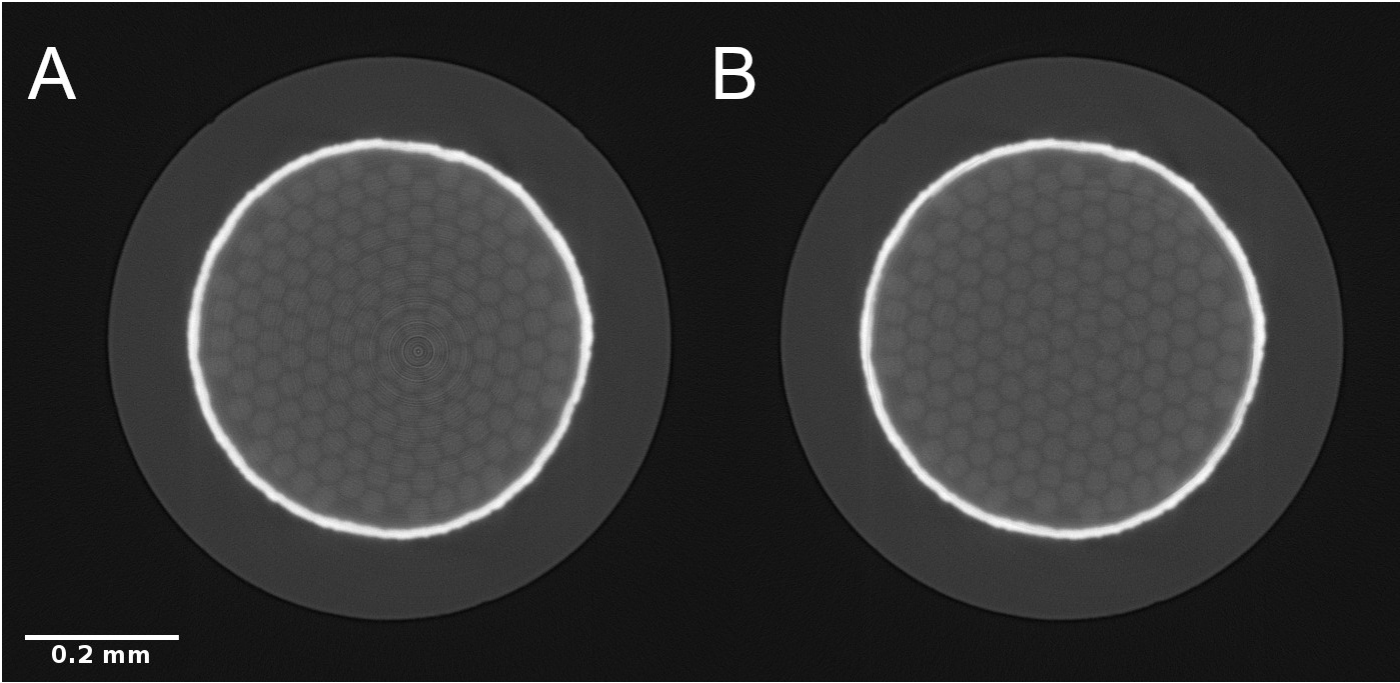
Reconstructed SXCT slice (detail) through a Nb _3_Sn superconducting wire without ( **A**) and with ( **B**) stripe artifact correction pre-processing step. Scale bar: 0.2 mm.

Figure 7A shows the results of CT reconstruction of a high X-ray energy scan of a sample of Nb _3_Sn (Niobium-Tin) superconducting wire with a Tantalum barrier ^31^. Without stripe artifact correction, the reconstructed image is affected by ring artifacts. In Figure 7B, a pre-processing step was applied to the normalized sinogram in order to remove full and partial stripe artifacts. Stripe removal was performed using Nghia Vo’s approach ^1^ using the *remove_stripe_based_sorting* method implemented in TomoPy. Both reconstructions were performed with TomoPy’s implementation of the *gridrec* algorithm ^32^. All steps were performed in Alrecon.

## Discussion

We have presented Alrecon, an open-source, pure Python web application that provides a modern and reactive graphical user interface for the reconstruction of synchrotron X-ray computed tomography scans using TomoPy. Alrecon is designed with a general beamline user in mind, and its use does not require programming skills. At the same time, Alrecon provides unique features that can benefit not only general users, but also experienced beamline staff. These include:

- Control over the sinogram portion to be loaded and processed
- Module for the optimization of the tomographic center of rotation for CT reconstruction
- Module for the correction of stripe artifacts from the sinogram
- Different CT reconstruction algorithms, including iterative methods
- GPU-accelerated CT reconstruction using TomoPy and ASTRA
- Output controls for the correction of the reconstructed volume data range and integer conversion
- Possibility to schedule reconstruction jobs on HPC cluster
- Automatic logging of reconstruction jobs to Google spreadsheet

### Advantages of Alrecon

The possibility to render multidimensional images directly within a Python notebook using Jupyter Widgets has motivated the development of tools similar to Alrecon such as TomoPyUI: a Jupyter-based TomoPy reconstruction graphical user interface ^33^. Alrecon profits from the advantages provided by Python and ipywidgets, but uses Solara to deploy a web application with a clean, intuitive and user-friendly layout.

The use of Solara which, in turn, is based on React, makes Alrecon reactive and trustworthy, while keeping the source code extremely simple. Common beamline users can launch and control CT reconstruction tasks from a simple web page, focusing exclusively on the optimization of the reconstruction parameters. While they do so, Alrecon takes care of scheduling jobs on the beamline HPC facility and keeps an automatic online log of the submitted jobs.

The Alrecon user interface is designed with the specific goal to enable seamless inspection of both raw sinogram data, and reconstructed 3D images, immediately after the completion of SXCT scans. The possibility to attempt CT reconstruction of a dataset directly after data has been captured can be crucial to optimize experimental conditions and data collection parameters. Often, settings such as the X-ray beam energy, and the detector position or configuration, influence the quality and contrast of the SXCT image, determining the possibility to visualize and segment features of interest. Visualizing CT reconstructions immediately after the scan is the best way to obtain feedback on the quality of the experiment and allows quick identification of problems.

Alrecon exploits two wide-spread image visualization and analysis tools: napari and ImageJ. The pure python implementation of the Alrecon reconstruction environment allows the use of napari for 3D volume data visualization. Sinograms and reconstructed datasets living in multidimensional numpy arrays can be displayed by napari within Python, removing intermediate write-to-disk operations. At the same time, a launcher allows open results written to disk using ImageJ, which provides the user an extensive set of image analysis capabilities.

The simplicity of the source code, together with the modularity provided by Solara, makes Alrecon easily customizable. We have developed a HPC settings card that can be used to customize the Slurm job files written by Alrecon ( Figure 4B). We expect this to help different laboratories in the adoption and set-up of Alrecon for use on their HPC facility. Other examples of possible customization include the use of beamline-specific input/output methods, depending on the HDF5 file architecture in use, and on the desired file format for the reconstructed datasets, or the use of Alrecon with CT reconstruction software other than TomoPy. Among the future developments that we envision, is the possibility to perform CT reconstructions using Tomocupy, a python package specifically developed for efficient GPU reconstruction implementing both 32-bit and 16-bit arithmetics ^12^.

Finally, the Alrecon Solara app can be run within Jupyter (e.g. as a Jupyter Notebook) or as a standalone server. The Alrecon reconstruction environment can be set up on a local workstation, or on a server accessing computing resources on demand. The possibility to process subsets of collected sinograms allows Alrecon to run on a machine with minimal computing resources.

## Data Availability

Zenodo: Underlying data for ‘Alrecon: computed tomography reconstruction web application based on Solara’, ‘Synchrotron X-ray Computed Tomography scan of a wasp’, https://doi.org/10.5281/zenodo.10075277 ^30^ This project contains the following underlying data: bee_yazeed-20231001T170032.h5. (SXCT scan of a wasp performed at the ID10-BEATS beamline of SESAME. The HDF5 file contains raw data and metadata following the Scientific Data Exchange standard.) SESAME_wasp_yazeed.avi. (3D video rendering of phase-contrast CT reconstruction of bee_yazeed-20231001T170032. The dataset was reconstructed using Alrecon. The video was created using ORS Dragonfly.) Data are available under the terms of the Creative Commons Attribution 4.0 International license (CC-BY 4.0)

## Software availability

Source code available from: https://github.com/gianthk/alrecon/tree/master

Archived source code available from: https://zenodo.org/records/10535212 ^33^

License: MIT License.
