# Supplementary material for: Alrecon: computed tomography reconstruction web application based on Solara
Source: Open Res Eur. 2024 May 28;4:54. Originally published 2024 Mar 18. [Version 2] doi: 10.12688/openreseurope.16863.2 (PMC11109687; doi:10.12688/openreseurope.16863.2)
Supplement: Peer review history

# Reviewer report: Reviewer response for version 2

**DOI:** 10.21956/openreseurope.19270.r48211

**Published:** 2025-01-03

**Authors:** Vânia Vieira Estrela

**Affiliations:** 1 Federal Fluminense University, Niterói, Brazil

Firstly, commendations to the authors for presenting Alrecon, a Python-based web application GUI that reconstructs synchrotron XCT that functions as a graphical user interface for widely used tools within the community, including TomoPy and ASTRA. The application offers streamlined access to a conventional processing pipeline, encompassing data loading, pre-processing, reconstruction, and storing functionalities with compression and information recording. Scalability is achieved by executing cluster computations using the SLURM task scheduler to confer scalability to Alrecon. The user can examine the data at every processing level via external apps like ImageJ and Napari, which can start from the application.

 The following remarks are more related to the whole cyber-physical system (CPS) as perceived by users like health care professionals and biomedical/computer engineers while bearing in mind the project nature, the reviewers' remarks, and the authors' replicas. SXCT, despite some caveats in nuclear engineering, is quite good and promising in terms of extremely high resolution. Organ-to-cellular and organ-to-molecule scale imaging are crucial in neurological studies, the blood-brain barrier (BBB), and connectome mapping. These tasks are in their infancy, but they can be decisive in developing future medications targeting the brain, which more than justifies SXCT. Of course, other applications exist, but this is the genesis of the questions below.

1. What about the software scalability and its ability to incorporate other pieces of code or suites from other synchrotron- and non-synchrotron facilities via cloud computing under the assumption that cybersecurity (CS) and reliable data (e.g., protection via blockchain) are robust so the interface's problems will only come from detectors' data?
2. Alrecon leads to two huge computational software challenges: image pre-processing and 3-D, …, N-D reconstruction, where N is an integer greater than 3. For instance, super-resolution (SR) and hyperspectral imaging can be operational, considerably increasing the feature-vector dimension per voxel.
3. Can Alrecon handle and accommodate current and future number-crunching and storage-demanding algorithms adaptively? This question stems from the fact that pre-processing synchrotron images can partially correct the scanning results of an in vivo organism. The other part will be addressed by nuclear equipment specialists working with photon-counting detectors (PCD) in place of energy-integrating detectors (EID) to enhance SXCT imaging, which will eventually affect the data pre-processing step.
4. As is known, motion can affect SXCT even if the subject is still, and again, this is a problem that can be partially corrected through software. Here, Dr. E. Pasca's comments and Alrecon's design are sources of insights. People who develop hardware and software could extend the life cycle of their creations by leaving provisions for both hardware and software scalability and room for future virtual machines that will blend in new CPSs. Times are tough, and lots of paradigms coexist. Nevertheless, obsolescence should be postponed the most because of the costs involved in the detector's end and software engineering.
5. The questions above will also probably benefit from AI (e.g., deep learning)  and quantum machines to help obtain high-resolution, multi-, and hyperspectral spectral voxels adequate for more advanced non-destructive experiments, particularly in vivo.

In order to corroborate the issues mentioned above, the authors can peruse some of these references given with this report.

Is the rationale for developing the new software tool clearly explained?

Yes

Is the description of the software tool technically sound?

Yes

Are sufficient details of the code, methods and analysis (if applicable) provided to allow replication of the software development and its use by others?

Yes

Is sufficient information provided to allow interpretation of the expected output datasets and any results generated using the tool?

Yes

Reviewer Expertise:

Electronic and computer engineering, cyber-physical systems, Industry 5.0, multidimensional signal processing, biomedical engineering, multimedia, neuroinformatics

I confirm that I have read this submission and believe that I have an appropriate level of expertise to confirm that it is of an acceptable scientific standard.

## References

[1] : Guideline for software life cycle in health informatics. *iScience* .2022;25(12) : 10.1016/j.isci.2022.105534 105534 10.1016/j.isci.2022.105534 36437879 PMC9685384

[2] Systems and software engineering - Life cycle management. *International Standard* . Reference source

[3] : Sparse2Noise: Low-dose synchrotron X-ray tomography without high-quality reference data. *Comput Biol Med* .2023;165: 10.1016/j.compbiomed.2023.107473 107473 10.1016/j.compbiomed.2023.107473 37690288

[4] : Deep 3D reconstruction of synchrotron X-ray computed tomography for intact lungs. *Sci Rep* .2023;13(1) : 10.1038/s41598-023-27627-y 1738 10.1038/s41598-023-27627-y 36720962 PMC9889716

[5] : Multidimensional Analysis of the Adult Human Heart in Health and Disease Using Hierarchical Phase-Contrast Tomography. *Radiology* .2024;312(1) : 10.1148/radiol.232731 e232731 10.1148/radiol.232731 39012246 PMC11303834

[6] : Benchtop cone-beam X-ray fluorescence CT system with high sensitivity and high spatial resolution. *IEEE* . https://doi.org/10.1109/NSSMICRTSD49126.2023.10338068 10.1109/NSSMICRTSD49126.2023.10338068

[7] : Tracking cells in the brain of small animals using synchrotron multi-spectral phase contrast imaging. *HAL Open Science* . 10.1117/12.2580841 10.1117/12.2580841

# Reviewer report: Reviewer response for version 2

**DOI:** 10.21956/openreseurope.19270.r47665

**Published:** 2024-12-26

**Authors:** Luisa Vigorelli

**Affiliations:** 1 Università di Milano-Bicocca and INFN, Milano, Italy

In this paper, the authors present a new software, called Alrecon, developed for synchrotron computed tomography reconstruction. The software is realized using Solara, a tool that allow to build web apps based on pure Python language. The software is designed and created trying to build a graphical interface easy to understand and use even for those with less programming experience, separating the tools for the different reconstruction phases. Synchrotron users will be able to use this tool for tomographic reconstructions immediately after acquisitions and verify step by step the quality of the reconstruction, also thanks to the use of visualization tools such as ImageJ or Napari. In this way, the user is guided through all the work phases, having the possibility of adopting the best parameters to obtain high-quality reconstruction (centre of rotation research, ring artefacts correction). The software gives the possibility of viewing and reconstructing both absorption and phase contrast images, using different phase retrieval algorithms for the latter.

 The authors provide a test database for users in order to become familiar with the software and the link for the source code. The authors report also some consideration about aspects and functionalities that can be integrated as future development of the software.

 Overall, the paper is well written and easy to read and understand, I certainly recommend it for approval. 

- This new tool seems very promising; in the current configuration, could it be employed for non-synchrotron CT reconstruction too? Do some code parts have to be added/modified, for example beam geometry? (just a curiosity)
- Does it make sense to add a tool for beam hardening correction in AIrecon (if not already present)?  

**Is the rationale for developing the new software tool clearly explained?**

 The authors give the rationale description in the Introduction section. Some already developed toolkits are optimized for application on stand-alone GPU workstations, however with some drawbacks, as data dimensions, manipulation and storage. Moreover, more than one software or tool are often use for CT reconstruction, sometimes needing some expertise and resulting time consuming. Alrecon can be used by SXCT beamline staff, as well as researchers or students lacking programming experience.

**Is the description of the software tool technically sound?**

 In the paper the software construction is described, with single procedures and the general CT reconstruction pipeline. All the parts are well described, with detailed figures. The authors do not report the architecture of the web application or provide code examples.

**Are sufficient details of the code, methods and analysis (if applicable) provided to allow replication of the software development and its use by others?**

 The authors describe principally the functional and graphical aspects of the software rather than the baseline code; however, this make sense as the aim of the paper is to present a new software thought for enabling users to access functionality from multiple packages. They also describe the single modules reporting some application examples, useful for a better understanding.

**Is sufficient information provided to allow interpretation of the expected output datasets and any results generated using the tool?**

 The authors provided some examples of different software functionalities, showing the potential of the tool; the results are presented with explicative images and parameters.

Is the rationale for developing the new software tool clearly explained?

Yes

Is the description of the software tool technically sound?

Yes

Are sufficient details of the code, methods and analysis (if applicable) provided to allow replication of the software development and its use by others?

Partly

Is sufficient information provided to allow interpretation of the expected output datasets and any results generated using the tool?

Yes

Reviewer Expertise:

Computed tomography, material science

I confirm that I have read this submission and believe that I have an appropriate level of expertise to confirm that it is of an acceptable scientific standard.

# Reviewer report: Reviewer response for version 2

**DOI:** 10.21956/openreseurope.19270.r48204

**Published:** 2024-12-11

**Authors:** Tristan van Leeuwen

**Affiliations:** 1 Centrum Wiskunde & Informatica, Amsterdam, The Netherlands

The authors introduce a web-based front-end for tomographic reconstruction of Synchrotron X-ray CT data. The tool combines several steps in the typical data-processing workflow in a way that is designed to be easily accessible for beamline scientists. The functionalities include preprocessing, reconstruction, and some post processing and scheduling of compute-heavy jobs to a connected HPC cluster as well as logging of the experimental parameters and processing steps. Overall, it seems a very relevant tool that can be used to address the gap between experimentalists, who collect the data, imaging scientists, who interpret the data, and computational scientists, who develop the algorithms. As such, it is a great step forward in making such involved workflows reproducible.

 Generally, the paper is well-written and serves as a good overview of the corresponding software. I don't think the paper is meant as a step-by-step tutorial but it seems to contain sufficient detail to get started. I have not tried to run the accompanying examples nor tried to install the code. so I cannot say it the steps in the paper and documentation suffice. I was also wondering if there is a way to user another cloud-based service for logging if one does not want to rely on Google. I am not an expert, but I can imagine that for some users / applications this may cause legal problems if meta-data related to their project is stored in certain geographical locations.

 I have a few minor textual remarks that could help improve readability.

 1. The abstract is rather verbose and contains quite a lot of technical jargon (Radiograph, Radon transform. Solara). as well as in my view unnecessarily verbose explanations (such as mentioning that the images are written to disk).

 2. On page 3 a few examples of artifacts are mentioned (stripes, rings); it would be nice to have an illustration of this to help the reader.

 3. On page 4, statement of need, it is mentioned that Alrecon is user-friendly; this seems a subjective statement. I understand that it is design that way, but has it's user-friendliness been evaluated? if so, how?

 Similarly, the 'flawless log' seems strange; a flawed log would be useless. Perhaps replace by detailed log? Even then, what does this mean? Which details are logged?

 4. On page 4 Pre-processing and reconstruction... it is mentioned that the sinogram is loaded as a 3D numpy array -- is this crucial information? If so, what is the type used? float32? Otherwise, just replace with '3D array' or 'loaded in memory'?

 5. On page 10 it states 'the simplicity of the source code', which is a subjective term. I would replace it by 'the source code has been designed to facilitate customisation' or such and refer to detailed documentation of the API.

Is the rationale for developing the new software tool clearly explained?

Yes

Is the description of the software tool technically sound?

Yes

Are sufficient details of the code, methods and analysis (if applicable) provided to allow replication of the software development and its use by others?

Partly

Is sufficient information provided to allow interpretation of the expected output datasets and any results generated using the tool?

Yes

Reviewer Expertise:

Computational Imaging

I confirm that I have read this submission and believe that I have an appropriate level of expertise to confirm that it is of an acceptable scientific standard, however I have significant reservations, as outlined above.

# Reviewer report: Reviewer response for version 2

**DOI:** 10.21956/openreseurope.19270.r46389

**Published:** 2024-12-10

**Authors:** Chang-Chieh Cheng

**Affiliations:** 1 Information Technology Service Center, National Yang Ming Chiao Tung University (Ringgold ID: 34914), Hsinchu, Taiwan

This paper introduces Alrecon, a web-based system with an intuitive graphical user interface designed to support synchrotron X-ray computed tomography (SXCT). By integrating widely used tools such as TomoPy, ASTRA, napari, and ImageJ, it offers a streamlined solution for SXCT workflows. Alrecon also incorporates DXChange and Slurm, enabling users to schedule tomographic reconstruction tasks on high-performance computing (HPC) systems with minimal programming expertise. Built with Python and released as open source, the system is highly customizable and extensible.

 The paper is well-structured and presents its ideas in a logical and coherent manner. However, I have several recommendations:

 1. For Figures 6 and 7, consider including enlarged views for each subimage to emphasize regions affected by significant COR or stride artifacts.

 2. I suggest that the authors provide a series of images demonstrating how napari can be used to visualize a reconstructed volume.

 3. COR and ring artifacts are common issues in SXCT. Many methods for correcting these artifacts have been proposed. I recommend that the authors cite recent works, such as References 1-4, to provide a more detailed background. Additionally, it would be helpful to include an example illustrating how Alrecon could be extended to incorporate external programs for COR or ring artifact correction.

 Refereces: 

 [Ref 1], [Ref 2], [Ref 3] and [Ref 4]

Is the rationale for developing the new software tool clearly explained?

Yes

Is the description of the software tool technically sound?

Yes

Are sufficient details of the code, methods and analysis (if applicable) provided to allow replication of the software development and its use by others?

Yes

Is sufficient information provided to allow interpretation of the expected output datasets and any results generated using the tool?

Yes

Reviewer Expertise:

Image processing, computational imaging, visualization

I confirm that I have read this submission and believe that I have an appropriate level of expertise to confirm that it is of an acceptable scientific standard.

## References

[1] : Correction of center of rotation and projection angle in synchrotron X-ray computed tomography. *Sci Rep* .2018;8(1) : 10.1038/s41598-018-28149-8 9884 10.1038/s41598-018-28149-8 29959398 PMC6026166

[2] : Deep Learning-based Inaccuracy Compensation in Reconstruction of High Resolution XCT Data. *Sci Rep* .2020;10(1) : 10.1038/s41598-020-64733-7 7682 10.1038/s41598-020-64733-7 32376852 PMC7203197

[3] : Structure-aware guided filtering for a ring artifact correction in synchrotron x-ray microtomography. *Appl Opt* .2023;62(28) : 10.1364/AO.501832 7400-7410 10.1364/AO.501832 37855508

[4] : Deep-learning-based ring artifact correction for tomographic reconstruction. *J Synchrotron Radiat* .2023;30(Pt 3) : 10.1107/S1600577523000917 620-626 10.1107/S1600577523000917 36897392 PMC10161896

# Reviewer report: Reviewer response for version 1

**DOI:** 10.21956/openreseurope.18220.r39375

**Published:** 2024-05-07

**Authors:** Edoardo Pasca

**Affiliations:** 1 Science Technology Facilities Council, Swindon, UK

The authors present Alrecon an OS python web application GUI for reconstruction of synchrotron XCT. The application serves as a GUI for popular tools in the community such as tomopy. The app provides simplified access to a standard processing pipeline with data loading, pre-processing (normalisation, centre of rotation correction, ring artifact/stripe remover, phase retrieval filter), reconstruction (with tools provided by the underlying packages tomopy and ASTRA) and, saving capabilities with compression and meta data logging. Scalability is obtained by being able to run the computation on clusters via SLURM job scheduler. At each stage of the processing the user is able to inspect the data via external applications such as ImageJ and napari which can be launched from the application itself.

- Is the rationale for developing the new software tool clearly explained?

The authors introduce Alrecon is a GUI for synchrotron XCT which relies on other OS packages used by the community, such as tomopy, ASTRA, dxchange and leverages on scalable processing via SLURM.

 The need for developing the UI is well explained: processing the XCT data often requires multiple software and expert users. The UI is designed to provide a simple intuitive tool for the XCT pipeline.

- Is the description of the software tool technically sound?

The authors describe the UI and the tools it provides in relative detail. They do not describe the architecture of the web application or provide code snippets rather they describe the pipeline it implements and they substantiate it with 3 with use cases.

- Are sufficient details of the code, methods and analysis (if applicable) provided to allow replication of the software development and its use by others?

As the presented software tool is a GUI, the authors focus the description on the functional and graphical aspects rather than the underlying code. The authors exploit 3 use cases to describe the phase retrieval, centre of rotation and ring/stripe removal procedures that are available in Alrecon. For each case, they provide detailed configuration of the procedure with links to the data utilised for one of the use cases.

- Is sufficient information provided to allow interpretation of the expected output datasets and any results generated using the tool?

Examples of output of the tool are described with figures and parameters. This is considered sufficient as the aim of the publication is to discuss a GUI that allows users access functionality from other packages rather than presenting the actual functionality or technical details of the application.

Is the rationale for developing the new software tool clearly explained?

Yes

Is the description of the software tool technically sound?

Yes

Are sufficient details of the code, methods and analysis (if applicable) provided to allow replication of the software development and its use by others?

Yes

Is sufficient information provided to allow interpretation of the expected output datasets and any results generated using the tool?

Yes

Reviewer Expertise:

Computed tomography, software

I confirm that I have read this submission and believe that I have an appropriate level of expertise to confirm that it is of an acceptable scientific standard.

# Reviewer report: Reviewer response for version 1

**DOI:** 10.21956/openreseurope.18220.r39380

**Published:** 2024-05-06

**Authors:** Viktor Nikitin

**Affiliations:** 1 Argonne National Laboratory, Lemont, Illinois, USA

In this paper, the authors report their recently developed software package AIrecon for CT data reconstruction and visualisation. The main feature of the package is its user-friendly web interface based on Solara, which allows users to build web apps from pure Python using ipywidgets or a React-like API on top of ipywidgets. This web interface makes the package usable for experienced beamline staff, researchers, and students without programming experience. The interface allows for selecting reconstruction parameters, starting visualisation packages (ImageJ and Napari), submitting a job to an HPC cluster, and other functionality. Reconstruction methods are called from publicly available and already well-functioning TomoPy and Astra packages.

 The AIrecon package is publicly available at https://github.com/gianthk/alrecon/tree/master and can be easily installed and tested. The tests can be performed on an experimental h5 dataset acquired at ID10 beamline of SESAME synchrotron. The authors provided access to these data via Zenodo.

 The paper is well-structured and easy to read, and I certainly recommend it for approval. There are just a couple of comments:

1. It would be nice to mention Solara in the Introduction since not all people are familiar with it.
2. I recommend mentioning real-time reconstruction tools like Recast3D (Buurlage, et al., 2018 [Ref 1]) and TomoStream (Nikitin, et al., 2022 [Ref 2]) and commenting on whether something like that can be implemented within AIrecon. Also, in the current version, ImageJ is called from the local computer.
3. There is an ImageJ working in the browser at https://ij.imjoy.io/. Does it make sense to adapt it for AIrecon? Just a recommendation.

Is the rationale for developing the new software tool clearly explained?

Yes

Is the description of the software tool technically sound?

Yes

Are sufficient details of the code, methods and analysis (if applicable) provided to allow replication of the software development and its use by others?

Yes

Is sufficient information provided to allow interpretation of the expected output datasets and any results generated using the tool?

Yes

Reviewer Expertise:

Computed tomography, software development

I confirm that I have read this submission and believe that I have an appropriate level of expertise to confirm that it is of an acceptable scientific standard.

## References

[1] : Real-time quasi-3D tomographic reconstruction. *Measurement Science and Technology* .2018;29(6) : 10.1088/1361-6501/aab754 10.1088/1361-6501/aab754

[2] : Real-time streaming tomographic reconstruction with on-demand data capturing and 3D zooming to regions of interest. *J Synchrotron Radiat* .2022;29(Pt 3) : 10.1107/S1600577522003095 816-828 10.1107/S1600577522003095 35511014 PMC9070713
